# Salvia Miltiorrhiza Ameliorates Liver Fibrosis by Activating Hepatic Natural Killer Cells *in Vivo* and *in Vitro*

**DOI:** 10.3389/fphar.2018.00762

**Published:** 2018-07-16

**Authors:** Yuan Peng, Tao Yang, Kai Huang, Li Shen, Yanyan Tao, Chenghai Liu

**Affiliations:** ^1^Institute of Liver Diseases, Shuguang Hospital Affiliated to Shanghai University of Traditional Chinese Medicine, Shanghai, China; ^2^Department of Cardiology, Shuguang Hospital Affiliated to Shanghai University of Traditional Chinese Medicine, Shanghai, China; ^3^Shanghai Key Laboratory of Traditional Chinese Clinical Medicine, Shanghai, China; ^4^Key Laboratory of Liver and Kidney Diseases, Ministry of Education, Shanghai, China

**Keywords:** salvia miltiorrhiza (SM), natural killer (NK) cells, hepatic stellate cell (HSC), carbon tetrachloride (CCl_4_), liver fibrosis

## Abstract

Natural killer (NK) cells are known for their ability to kill activated hepatic stellate cells (HSCs), which has been confirmed both in patients and animal models. But the killing function is depressed in period of advanced liver injury. Salvia Miltiorrhiza (SM), a Chinese herbal medicine for invigorating blood circulation and eliminating stasis, is widely used to treat liver fibrosis in clinic. Nevertheless, the immunological mechanism remains unclearly. Here, we put forward the hypothesis that the anti-fibrotic effect of SM is concerned with boosting the activation of hepatic NK cells. Liver fibrosis was induced with carbon tetrachloride (CCl_4_) and effects of SM on NK cells and HSC (JS-1 cell line, HSC) were investigated *in vivo* and *in vitr*o. Hepatic NK cells were isolated from C57BL/6 mice, and pre-incubated with SM before they were co-cultured with HSCs. We found that SM increased frequency of NK cells, enhanced activities of NKG2D and Nkp46 on NK cells and inhibited activation of HSCs *in vivo* and *in vitro*. SM could promote the activities of NK cells by increasing the expressions of NKG2D and IFN-γ before or after co-cultured with HSCs *in vitro*. Besides, SM could partially antagonize ASGM-1-induced NK cell depletion and enhance the cell activities to inhibit HSCs activation *in vitro*. Therefore, our work provided a new insight into the anti-fibrotic mechanism that SM could enhance the activities of NK cell to reduce liver fibrosis *in vivo* and *in vitro*.

## Introduction

Liver fibrosis is characterized by the diffuse nodular regeneration surrounded with dense collagen deposition and collapse of liver structures. Liver fibrosis leads to cirrhosis, hepatocellular carcinoma, and ultimately liver failure, which is accompanied by significant morbidity and mortality (Liu et al., 2015). Fibrotic diseases account for up to 45% of deaths in the developed countries. However, effective antifibrotic therapies are lacking (Mehal et al., 2011).

During chronic liver injury, various factors, such as viruses, toxins and drugs, contribute to the activation of hepatic stellate cells (HSCs) (Friedman, 2008). Activated HSCs transform into proliferative, fibrogenic, and contractile myofibroblasts and secrete ECM. Therefore, inhibiting the activation of HSCs is a key step in the treatment of liver fibrosis. So far, no pharmaceuticals or other medical treatments have been approved by Food and Drug Administration (FDA) yet to treat liver fibrosis patients.

Natural killer (NK) cells are important components of the innate immune system, which account for 10–20% of the total intra-hepatic lymphocytes in mouse livers (Tian et al., 2013). The functions of NK cells are to kill target cells and produce a variety of cytokines, such as IFN-γ, perforin, and trail. Whether NK cells aim to kill the target cells or not depends on the NK cells and their interactions with the corresponding ligands that are expressed on the surface of the target cells (Raulet, 2003; Lanier, 2005). The inhibitory receptors, such as Ly49A and CD94/NKG2, interact with the inhibitory ligands (e.g., MHC-I) expressed on the target cells to suppress NK cell function. Simultaneously, the stimulatory receptors, including NKG2D, NKp46, NKp30 and NKp44, interact with the stimulatory ligands (e.g., RAE-1) which are expressed on the surface of the target cells and promote NK cell activities. Among these, NKG2D is the most well-defined receptor which binds its ligand RAE-1 that is expressed on the target cells and subsequently promotes NK cell activation (Melhem et al., 2006).

Accumulating evidence suggests that NK cells play an important role in controlling liver fibrosis and cirrhosis. NK cells killing of activated HSCs was reported in both human and animal experiments (Radaeva et al., 2006; Varchetta et al., 2012). However, through several established mechanisms, the NK-cell versus activated HSC effect is attenuated as the disease inexorably progresses. In fact, the anti-fibrotic effect of NK cells was suppressed during advanced liver injury (Jeong et al., 2011), which contributed to the progression of liver fibrosis. Therefore, restoring and promoting the activities of NK cells, in part, might be one of the important ways for treatment of liver fibrosis.

The dried root of *Salvia miltiorrhiza* Bunge (Lamiaceae) (SM), often referred as Danshen in China, is a very popular medicinal plant that has been extensively applied to treat various diseases. Because of its characteristic of improving blood circulation, it is widely used to treat heart diseases and cerebrovascular diseases, either alone or in combination with other Chinese herbal medicines (Lam et al., 2006; Zhou et al., 2011). Besides, SM has also been shown to reverse liver fibrosis in carbon tetrachloride induced liver fibrosis in rats, with a better impact on reducing levels of transforming growth factor-β1, procollagens I and III (Wasser et al., 1998). Nevertheless, little is known about how SM protects against liver fibrosis and whether an immunological mechanism may be involved.

In this study, we aimed to explore whether the anti-fibrotic effect of SM was related to its regulation of NK cell activities. And we also attempted to analyze how far SM modified the interactions between NK cells and HSCs. The understanding of SM-mediated immunoregulatory effect on NK cells might provide pivotal insights into cellular and molecular mechanisms for liver disease progression.

## Materials and Methods

### Reagents

Analytical reagent grade carbon tetrachloride (CCl_4_) was obtained from Sinopharm Group, Co, Ltd. (Shanghai, China). Chromatography grade regents for drug identification were purchased from Merck (Darmstadt, Germany). All other chemicals and solvents of analytical grade were obtained from Sangon Biotech (Shanghai), Co., Ltd.

### Drug Preparation and Identification

Radix *Salviae Miltiorrhizae* (SM) was purchased from Shanghai Shyndec Pharmaceutical, Co., Ltd. (Shanghai, China). SM extract was prepared as follows: 1000 g of SM were heated under reflux with 90% ethanol for 1.5 h and then were filtered by the 120 mesh. The ethanol was recovered and the filtrate was concentrated to a thick extract. Subsequently, the residue was decocted with water for 1 h and was filtered by the 120 mesh. Ultimately, the filter and the above thick extract were combined and concentrated under vacuum at 50°C and then dried by lyophilization to afford the extraction of SM (120 g). The extract of SM was identified by Dr. Tao Yang, according to the Pharmacopoeia of the People’s Republic of China (2015). The voucher specimen (No. 20160428) was deposited at Shuguang Hospital affiliated to Shanghai University of Traditional Chinese Medicine (Shanghai, China).

**FIGURE 1:**
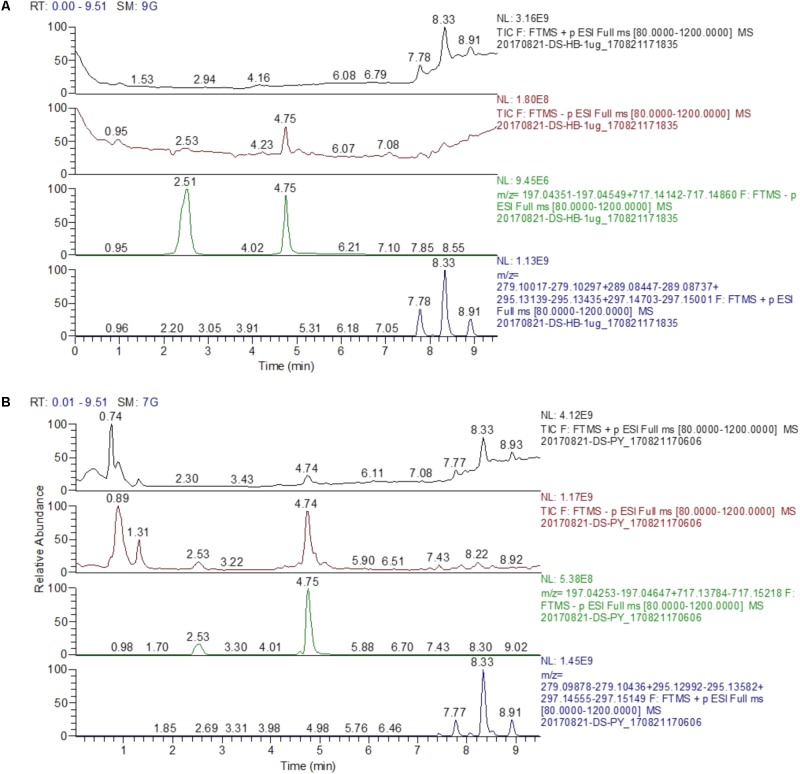
The chromate graphic profile of mixed standard and SM extract. **(A)** The chromatogram of mixed standard. **(B)** The chromatogram of SM extract; Peak retention time (TR): 2.51 min, tanshinol; 4.75 min, salvianolic acid B; 7.78 min, dihydrotanshino; 8.33 min cryptotanshinon; 8.91 min, tanshinone IIA [Stationary phase: waters acquity HSS T3 (100 mm × 2.1 mm, 1.8 μm); mobile phase: aqueous 0.1% formic acid (I) and acetonitrile (II) s 0.1% formic acid (I) and acetonitrile (II) (0–2 min, 10% II; 2–9 min, 10–95% II)].

To control the SM extract quality, the major bioactive components were carried out qualitative and quantitative analysis by chromatography-quadrupole/electro static field orbitrap high resolution mass spectrometry (UHPLC-Q/Exactive). The chromatographic profile of the extract was shown in **Figure 1**. The analyses were performed with a UHPLC-Q/Exactive system (Thermo Fisher, San Jose, CA, United States) equipped with a quaternary gradient pump, an autosampler and a quadrupole/electrostatic field orbitrap high resolution mass spectrometry detector. The components were eluted with a gradient system consisting of aqueous 0.1% formic acid (I) and acetonitrile (II) (0–2 min, 10% II; 2–9 min, 10–95% II). Otherwise, the contents of tanshinol, salvianolic acid B, dihydrotanshino, cryptotanshinon, and tanshinone IIA were detected by UHPLC-Q/Exactive method, and were respectively 5.48, 48.9, 0.045, 0.91, and 0.79 μg/mg in the extracts.

### Animals

Male C57BL/6 mice weighting 18–20 g, Specific-Pathogen-Free (SPF) level, were obtained from Shanghai Laboratory Animal Center, Chinese Academy of Sciences (Shanghai, China). All mice were housed under controlled temperature (22°C), humidity (55%), and lighting (12-h artificial light and dark cycle), with free access to tap water and mouse chow. The standard diet pellets contained not less than 20% protein, 5% fibers, 3.5% fats, and 6.5% ash and vitamins mixture. All the animal experiments were approved by the Committee on the Care and Use of Live Animals for Teaching and Research of the Shanghai University of Traditional Chinese Medicine (Approval Number: SZY201710014), and the procedures were performed according to the guideline of this committee.

### Cell Lines

JS-1 cell line, a spontaneously immortalized murine HSC, was a gift from Prof. Jinsheng Guo (Division of Digestive Diseases, Zhongshan Hospital, Department of Internal Medicine, Shanghai Medical College, Fudan University, Shanghai, China). JS-1 cells were cultured in Dulbecco’s modified Eagle’s medium (DMEM) supplemented with 10% fetal bovine serum (FBS, Gibco), 100 units/ml of penicillin, and 100 units/ml of streptomycin. All the cells were grown in a humidified incubator at 37°C in a 5% CO_2_ atmosphere.

### Experimental Design

All the mice were randomly divided into four experimental groups: normal control (*n* = 10), SM control (*n* = 10), model control (*n* = 10), SM 1.5 g/kg treatment (*n* = 10), and SM 3.0 g/kg treatment group (*n* = 10). Mice in model control, SM 1.5 g/kg treatment and SM 3.0 g/kg treatment group were given 10% CCl_4_ intraperitoneally (*i.p.*) at a dose of 2 ml/kg, dissolved in olive oil, three times a week for 6 weeks (Radaeva et al., 2006), except the normal control and SM control group which received vehicle only. From the 3^th^-week of CCl_4_ injection, mice in SM control, SM 1.5 g/kg treatment and SM 3.0 g/kg treatment group were treated orally with SM at a daily dose of 1.5 and 3.0 g/kg of body weight seven times a week for 4 weeks respectively, while mice in normal control and model control were administered orally by vehicle. All mice were sacrificed 48 h after the last CCl_4_ injection. Serum and liver samples were harvested. For histological analysis, tissue specimens were fixed in buffered formalin and embedded in paraffin wax. Serum and liver samples were kept frozen at −70°C until assayed.

### Measurements of Serum Liver Functions and Hydroxyproline (Hyp) Content

Activities of serumalanine aminotransferase (ALT) and aspartate aminotransferase (AST) were quantitated using commercial kits following the instructions provided by the manufacturer (Nanjing JianCheng Bioengineering Institute, Nanjing, China), including the use of standardization. Hyp levels in liver tissues were measured by the method described by Jamall et al. (1981).

### Histopathological Assessment of Liver Tissue and Quantitation of Liver Fibrosis

After routine processing, liver sections of 4-μm thickness were stained with hematoxylin and eosin (H&E) for histological analysis or Sirius red staining for collagen deposition. Images were analyzed with a light microscope (Olympus BX40, Japan). Quantitation of liver fibrosis was performed according to the results of Sirius red staining. The relative fibrosis area was expressed as a % of the total liver area, which was analyzed based on 40 visual fields of 5 Sirius red-stained liver sections per mouse. Each field was obtained at 10× magnification with a light microscope (Olympus BX40), and then Alliance software was used for the data analysis. To evaluate the relative fibrosis area, the measured collagen area was divided by the net field area and then multiplied by 100. Subtraction of the vascular luminal area from the total field area yielded the final calculation of the net fibrosis area.

### Flow Cytometric Analysis

Immediately after the mouse livers were removed, cells were recovered by mechanical disruption, and immunostained for flow cytometric analysis. The mononuclear cells (MNCs) of liver sample were extracted with Percoll (GE Healthcare, Sweden). The cells were collected, washed twice in 0.2% bovine serum albumin (BSA) in PBS buffer (0.2% BSA-PBS) and resuspended in PBS buffer for counting cell numbers with Trypan blue stain (Gibco, United States). Anti-mouse CD16/32 (eBioscience, United States) was used as the purified blocking antibodies. Immuno-phenotyping was performed on the samples with the following anti-mice antibodies: anti-mouse NK1.1-APC, anti-mouse CD3-PE-Cy7, anti-mouse NKG2D-PE, anti-mouse NKp46-PE, PE Rat IgG1κ Isotype and PE Rat IgG_2a_κ Isotype (BD PharMingen, United States). Data were acquired on BD LSR Foretessa flow cytometer (BD Biosciences, United States) and analyzed by using Flowjo 7.6 software.

### Cell Sorting

For primary NK cells, single hepatic lymphocytes from 6- to 8-week-old C57BL/6 mice were labeled with PE-Cy7-conjugated anti-CD3, PE-conjugated anti-CD4, APC-conjugated anti-NK1.1 and APC-Cy7-conjugated anti-CD19 (BD PharMingen, United States). NK cells were sorted as cells that express NK1.1^+^CD3^-^ using a FACS Aria I flow cytometer (BD Biosciences, United States) and the purity was higher than >95%. After purification, NK cells were cultured in a 96 round bottom wells (Costar, Corning Incorporated, Corning, NY, United States) in complete RPMI 1640 medium supplemented with 10% (v/v) fetal serum (Gibco, United States), 200 mg/ml glutamine, 50 μg/ml gentamycin, 100 units/ml penicillin, 100 μg/ml streptomycin (Sangon Biotech, Shanghai, China) and interleukin-2 (IL-2, 10 U/ml, BD PharMingen, United States).

### Drug Incubation

SM was initially dissolved as concentrated stock solution in sterile PBS. For evaluating the effect of SM on NK cells *in vitro*, primary hepatic NK cells were treated with seven concentrations (3.125, 6.25, 12.5, 25, 50, 100, 200 μg/ml) of SM in the presence of IL-2 for 16 h. For estimating the effect of SM on JS-1 cells *in vitro*, JS-1 cells were incubated with three concentrations (12.5, 25, 50 μg/ml) of SM for 24 h. For NK cell depletion *in vitro*, primary NK cells were kept in Rabbit IgG (Isotype Control) or ASGM-1 (dilution of 1:1000 in PBS), or ASGM-1 (dilution of 1:1000 in PBS) plus SM (50 μg/ml) for 4 h. Then the cells were harvested according to the experiments. All the experiments were repeated at least three times using independent cell culture.

### Co-culture of NK Cells and HSCs

The two types of cells were cultured in a 96 flat bottom wells (Costar, Corning Incorporated, Corning, NY, United States) at different ratio in complete RPMI 1640 medium: NK cells alone (shown as ratio 1:0), JS-1 alone (shown as ratio 0:1) and NK cells: JS-1 cells at the ratio 50:1.

### Cell Viability Assay

For the adherent cells, cell viability assays were performed by using the Cell Counting Kit-8 (CCK8, Beyotime Biotechnology, Jiangsu, China), according to the manufacturer’s protocol. For the suspension cells, LIVE/DEAD^®^ Viability/Cytotoxicity Kit (Molecular Probes, Invitrogen, United States) was used to test cell viability.

### Reverse Transcription Quantitative Polymerase Chain Reaction (RT-qPCR)

For the frozen liver tissues, total RNA was extracted by using Trizol reagent (Sangon Biotech, Shanghai, China). RNA was then used as a template for reverse transcription into single-stranded cDNA with random primers and reagents contained in the Reverse Transcription Reagent Kit with gDNA Eraser, according to the manufacturer’s protocol (TaKaRa, Dalian, China). RT-qPCR was performed using SYBR^®^ Premix Ex Taq^TM^ (TliRNaseH Plus) (TaKaRa) and ViiA^TM^ 7 Real-Time PCR System (ABI, Carlsbad, CA, United States). For primary NK cells, the cDNA was synthesized by Power SYBR^®^Green Cells-to-Ct^TM^ Kit (Life Technologies, Foster City, CA, United States) including reagents and enzyme mixtures for RT-qPCR, without isolating RNA. The synthesized β-actin gene was amplified as an internal control, and the other primers used are listed in **Table 1**.

**Table 1:** RT-qPCR primers used in this study.

Gene	Forward (5′ → 3′)	Reverse (5′ → 3′)
NKG2D	GCA TTG ATT CGT GAT CGA AA	GCC ACA GTA GCC CTC TCT TG
IFN-γ	ACT GGC AAA AGG ATG GTG AC	TGA GCT CAT TGA ATG CTT GG
Trail	CCC TGC TTG CAG GTT AAG AG	GGC CTA AGG TCT TTC CAT CC
Perforin	GAT GTG AAC CCT AGG CCA GA	GGT TTT TGT ACC AGG CGA GA
Ly49A	TTC TGG AAT CCC TCA ACA GG	GAA GGA ACC ACG AGC TGA AG
α-SMA	ACT ACT GCC GAG CGT GAG ATT G	CGT CAG GCA GTT CGT AGC TCT T
RAE-1ε	GCT GCA GTT CAA GAC ACC AA	TCC ACT GAG CAC TTC ACG TC
β-Actin	TGA CGA GGC CCA GAG CAA GA	ATG GGC ACA GTG TGG GTG AC

### F-Actin Cytoskeleton Staining

JS-1 cells were grown in a 96-wells chamber and treated with 5 ng/ml transforming growth factor-beta1 (TGF-β1) and drugs. After 24 h, cells were fixed in 4% formaldehyde and permeabilized with 0.2% Triton X-100. F-Actin was stained with rhodamin-phalloidin (1:100) (Molecular Probes, Inc., Eugene, OR, United States) and the nucleus with DAPI, according to the manufacturer’s protocol. Cells were visualized with by CellomicsArrayScan VTI HCS Reader and data were analyzed by Cellomics Cell Health Profiling BioApplication Software.

### Immunocytochemistry

JS-1 cells cultured on 96 wells were washed with cold PBS twice and fixed with cold methanol: acetone (1:1) for 10 min on ice. After extensive washing with PBS three times, cells were permeated with 0.05% saponin for 15 min. The cells were then blocked with 5% BSA-PBS buffer for 30 min at room temperature before incubated with the alpha smooth muscle actin (α-SMA) (1:100, A2547, Sigma, United States) or Collagen I (1:200, ab34710, Abcam, United States) as the primary antibodies. Cells were then stained with Cy3 or FITC-conjugated secondary antibodies. After washing, the cells were double-stained with Hoechst 33258 (Beyotime Biotechnology, Jiangsu, China) to visualize the nuclei. Images were taken by CellomicsArrayScan VTI HCS Reader.

### Statistical Analysis

All data were analyzed by using PASW Statistics 18 software. Differences between the groups were assessed by non-parametric one-way analysis of variance. Values in the text are presented as mean ± standard deviation (SD). *P* < 0.05 was considered statistically significant.

## Results

### SM Treatment Attenuated CCl_4_-Induced Liver Injury and Fibrosis

**FIGURE 2:**
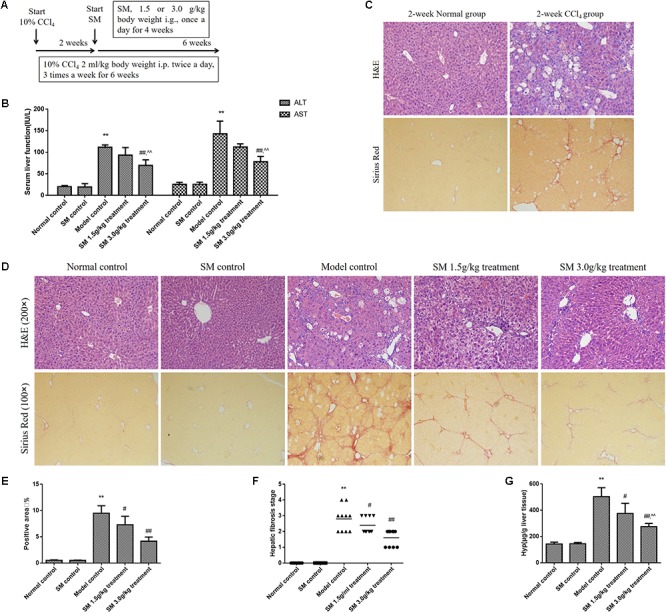
SM attenuated CCl_4_-induced liver fibrosis in mice. **(A)** Male mice were intraperitoneally injected with 10% CCl_4_, three times per week for 6 weeks, starting at 6 weeks of age. Two weeks after the CCl_4_ injection, once-daily treatment with SM extract 1.5 or 3.0 g/kg were begun. The normal control group received vehicle as control. Six weeks later, mice were sacrificed 48 h after the last CCl_4_ injection. **(B)** Liver fibrosis was examined by Sirius Red staining (magnification 100×) in the liver sections from mice injected with CCl_4_ or vehicle for 2 weeks. **(C)** Serum levels of serum ALT and AST were assayed by using commercial kits. **(D)** H&E- and Sirius Red- stained hepatic tissue sections from mice injected with CCl_4_ or vehicle for 6 weeks were examined under bright field microscope (H&E staining, magnification 200× and Sirius Red staining, magnification 100×). **(E)** Semi-quantification data for relative fibrosis levels were expressed as the % of total liver area, and the data were assessed by analyzing five fields of Sirius red stained liver sections per animal. Each field was acquired at 100× magnification, and data were acquired using Image-Pro Plus software. **(F)** Hepatic fibrosis stages of mice were analyzed according to the Knodell histological activity index. Fibrosis stages were determined by Ridit analysis. **(G)** Hyp contents were quantified from 100 mg liver samples and were measured by Jamall’s method. Values represent means ± SD (*n* = 10). ^∗^*P* < 0.05, ^∗∗^*P* < 0.01, versus normal control group; ^#^*P* < 0.05, ^##^*P* < 0.01, versus model control group. ^∧^*P* < 0.05, ^∧∧^*P* < 0.01, versus SM 1.5 g/kg treatment group.

The CCl_4_-treated mice were frequently used as an experimental model to study liver fibrosis (Mitra et al., 2014; Mukhopadhyay et al., 2014) (**Figure 2A**). To investigate the activities of NK cells on early stage (2-week-CCl_4_ administration) or advanced stage (6-week-CCl_4_ administration) of liver fibrosis, mice were injected with CCl_4_ for 2 or 6 weeks. Throughout the experiment, treatment with CCl_4_ successfully induced liver fibrosis and inflammation from the second week of CCl_4_ intraperitoneal injection without mortality. Levels of the serum transaminase from the model control group (6-week-CCl_4_ administration) were severer than those from the normal control group (**Figure 2B**). In addition, inflammation and collagen deposition in liver tissue were shaped after 2-week CCl_4_ injection (**Figure 2C**) and sharply exacerbated with the prolongation of CCl_4_ injection (**Figure 2D**). Therefore, model mice were treated with SM 1.5 or 3.0 g/kg from the third to the sixth week of CCl_4_-injection to observe the therapeutic effect on reversing liver fibrosis. After 4-week treatment of SM, ALT, and AST levels were significantly lower in mice receiving SM treatment, especially in SM 3.0 g/kg treatment group, compared to those in the 6-week CCl_4_ group (**Figure 2B**). Sections from the SM 1.5 and 3.0 g/kg treatment groups revealed less vacuolated cells and significantly improved portal inflammation and hepatocellular degeneration than those from the model control group (**Figure 2D**). Both the extent of hepatic fibrosis and the ratio of positive area of collagen deposition were significantly reduced (**Figures 2D–F**) and hepatic Hyp contents were decreased (**Figure 2G**) in SM-treated mice compared to those in the model control group. Besides, we tested whether a dose of 3.0 g/kg of animal body weight has toxic effects with a single treatment of SM. As shown in **Figure 2B**, treatment with SM for 4 weeks did not induce any significant changes in serum levels of ALT or AST. Moreover, H&E or Sirius red staining in SM control group all compared no difference with those in the normal control group. These results suggested that SM treatment could attenuate CCl_4_-induced liver injury and fibrosis, and the dosage of 3.0 g/kg of SM was found to perform more effectively against liver fibrosis.

### SM Promoted Activation of Intra-Hepatic NK Cells Inliver Fibrosis *in Vivo*

**FIGURE 3:**
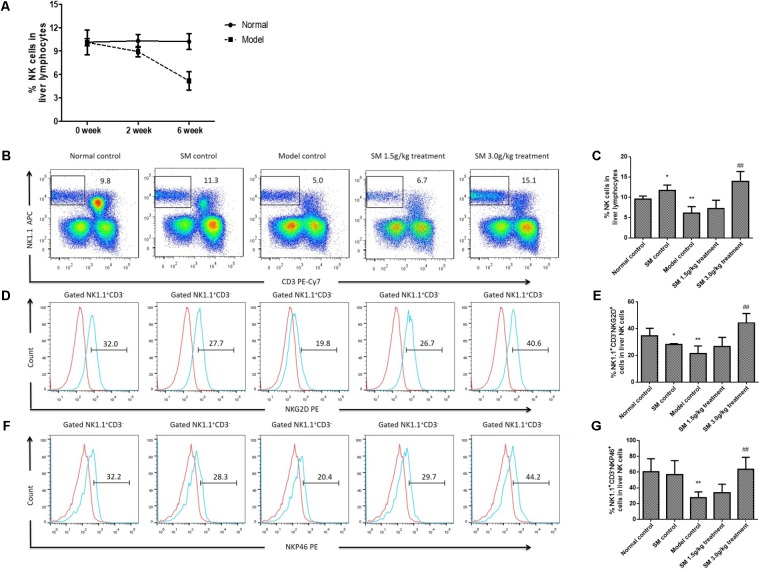
SM promotes activation of hepatic NK cells in CCl_4_-induced liver fibrosis *in vivo.* **(A)** Liver NK cells were examined from mice injected with CCl_4_ or vehicle for 2 or 6 weeks. Frequency of hepatic NK cells of normal or model mice after were assayed. **(B)** Liver lymphocytes were analyzed by FACS with APC-conjugated rat anti-mouse NK1.1 antibody and PE-Cy7-conjugated rat anti-mouse CD3 monoclonal antibody. **(C)** Frequency of NK1.1^+^CD3^-^ cells in liver lymphocytes were counted. **(D)** For NKG2D identification, a PE-conjugated rat anti-mouse NKG2D antibody was used. The expression of NKG2D from B was analyzed by FACS. **(E)** Frequency of NKG2D^+^ cells in liver NK1.1^+^CD3^-^ cells were counted. **(F)** Expression of NKp46 in NK1.1^+^CD3^-^ cells was quantified. **(G)** Frequency of NKp46^+^ cells in liver NK1.1^+^CD3^-^ cells were counted. Values represent means ± SD (*n* = 10). ^∗^*P* < 0.05, ^∗∗^*P* < 0.01, versus normal control group; ^#^*P* < 0.05, ^##^*P* < 0.01, versus model control group.

Nature killer cell exerting important anti-fibrotic activity on activated HSCs has been demonstrated by many studies both in animal models and patients (Jeong et al., 2006; Muhanna et al., 2011). Nevertheless, the anti-fibrotic effects of NK cells are suppressed during advanced liver injury, especially in progression of liver fibrosis (Jeong et al., 2011). We found frequency of intra-hepatic NK cells declined sharply significantly with the prolonged treatment of 4-week treatment of CCl_4_, which indicated that NK cells were decreased with the aggravation of liver fibrosis (**Figure 3A**). Based on the data above, we then investigated whether treatment with SM had beneficial effects on intra-hepatic NK cells during the development of liver fibrosis, since NK cells were suppressed in the advanced stages of liver fibrosis in mice (Jeong et al., 2011). By FACS analyses, the frequency of NK cells was up-regulated after SM treatment, especially after SM 3.0 g/kg treatment (**Figures 3B,C**). Besides, expressions of NKG2D and Nkp46 on NK cells after SM treatment (3.0 g/kg) were significantly increased compared to those in the model control group (**Figures 3D–G**). Interestingly, frequency of NK cells and their active receptors (NKG2D and NKp46) were significantly up-regulated in SM control group. Overall, activities of hepatic NK cells could be boosted by SM in a tendency with the dose-dependent manner.

### SM Promotes Activities of Primary NK Cells and Inhibits HSC Activation *in Vitro*

**FIGURE 4:**
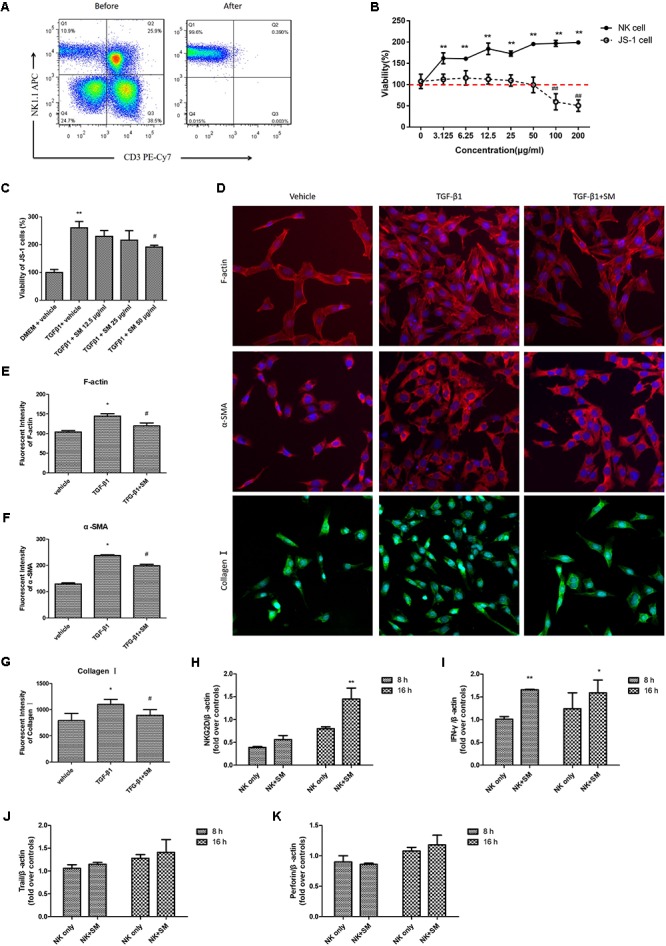
SM promotes activation of hepatic NK cells *in vitro.* **(A)** Hepatic NK cells were isolated from normal C57BL/6 mice livers, sorted by flow cytometry, and cultured in the presence of 10 IU/ml IL-2. The purity of primary NK cells was higher than 95%. **(B)** Cells were cultured in a 96-well plate at an appropriate density and (*incubated with 3.125–200 μg/ml of SM. For JS-1 cells, cytotoxicity was detected by CCK8 after incubation with SM for 24 h. For NK cells, cytotoxicity was analyzed by LIVE/DEAD^®^ Viability/Cytotoxicity Kit after incubation with SM for 16 h. **(C)** JS-1 cells were incubated with 5 ng/ml TGF-β1 for 24 h. Following cells were cultured with 12.5–50 μg/ml of SM for 24 h. The proliferation was analyzed by CCK8. The cells were represented in comparison with the model control (100%) and were shown as growth of the cells. **(D–G)** JS-1 cells were cultured in a 96-well plate at a density of 5,000 cells/well. Cells were incubated with 5 ng/ml TGF-β1 and 50 μg/ml of SM for 24 h. Images of F-actin, α-SMA and Collagen I were investigated by CellomicsArrayScan VTI HCS Reader. The nuclear were stained with DAPI. The image analysis of immunofluorescence used by Cellomics Cell Health Profiling Bio Application Software. ^∗^*P* < 0.05, ^∗∗^*P* < 0.01, versus vehicle. ^#^*P* < 0.05, ^##^*P* < 0.01, versus TGF-β1 group. **(H–K)** Primary hepatic NK cells were treated with 50 μg/ml of SM in the presence of IL-2, for a period of 16 h. mRNA levels of NKG2D **(H)**, IFN-γ **(I)**, trail **(J)**, and perforin **(K)** were quantified by RT-qPCR. ^∗^*P* < 0.05, ^∗∗^*P* < 0.01, versus NK only group.*)

Since SM could promote activation of intra-hepatic NK cells *in vivo*, we next observed whether SM treatment had analogous effects on NK cells *in vitro*. Primary hepatic NK1.1^+^CD3^-^ cells were isolated from the normal mice liver (**Figure 4A**), with the cell purity of over 95%. After identification by flow cytometry, NK cells were treated with seven concentrations (3.125, 6.25, 12.5, 25, 50, 100, and 200 μg/ml) of SM, in duplicate, for a period of 16 h. Surprisingly, the viability of NK cells was up-regulated with the increasing concentration of SM (**Figure 4B**). Meanwhile, we investigated the cell viability of JS-1 cells after continuous incubation of SM. As shown in **Figure 4B**, the survival ratio of JS-1 cells in incubations with 3.125–50 μg/ml of SM for 16 h exceeded 100%, which indicated no obvious toxic to the cells. Subsequently, JS-1 cells were incubated with 5 ng/ml TGF-β1 for 24 h to induce HSC activation. Then JS-1 cells were cultured with 12.5–50 μg/ml of SM. 24 h later, HSC activation could be inhibited by SM in a dose-dependent manner, and the maximum effect of SM was elicited at the concentration of 50 μg/ml (**Figure 4C**). Expectedly, expressions of F-actin, α-SMA, and Collagen I on activated HSCs were released after 50 μg/ml of SM incubation (**Figures 4D–G**). Thus, we examined SM at concentration of 50 μg/ml to assess the mechanisms of SM on NK cell functions in the following studies. After pre-incubation of SM for 8 or 16 h, expressions of NKG2D and IFN-γ were significantly up-regulated (**Figures 4H,I**). Expressions of trail and perforin were presented with no significant difference after SM incubation (**Figures 4J,K**).

**FIGURE 5:**
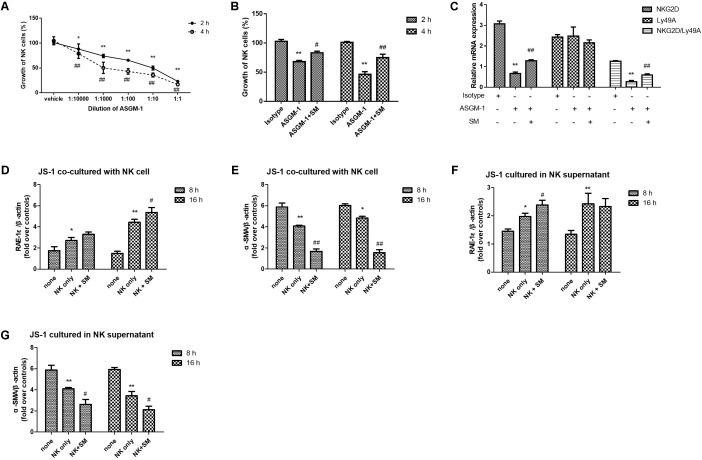
SM enhanced NK-cell activation on inhibiting HSCs *in vitro*. **(A)** Primary NK cells were isolated from normal C57BL/6 mice livers. NK cells were collected and incubated with ASGM-1 at concentrations of 1:1 to 1:10000 in PBS for 2 or 4 h respectively. The survival rate of NK cells were assayed by LIVE/DEAD^®^ Viability/Cytotoxicity Kit. **(B,C)** NK cells were exposed to ASGM-1 with dilution of 1:1000 in PBS for 2 or 4 h, and were treated with SM simultaneously. The survival rate of the cells **(B)** were tested. mRNA levels of NKG2D and Ly49A were quantified by RT-qPCR. ^∗^*P* < 0.05, ^∗∗^*P* < 0.01, versus isotype group; ^#^*P* < 0.05, ^##^*P* < 0.01, versus ASGM-1 group. **(D–G)** Primary NK cells were co-cultured with JS-1 cells for 8 and 16 h after they were pre-incubated with SM. Then NK cells and HSCs were separated. mRNA levels of α-SMA and RAE-1ε for HSCs were observed by RT-qPCR. ^∗^*P* < 0.05, ^∗∗^*P* < 0.01, versus none group; ^#^*P* < 0.05, ^##^*P* < 0.01, versus NK only group.

To confirm whether SM played a critical role on promoting activities of NK cells, we used an anti-ASGM-1 antibody to deplete the activities of NK cells in order to investigate the effect of SM on ameliorating the suppressive vitality of NK cell. Firstly, effects of ASGM-1 antibody on NK cells at concentrations of 1:1 to 1:10000 in PBS for 2 or 4 h were assayed. We found that the survival rate of NK cells declined with the increasing dilutions and incubation of ASGM-1 with dilution of 1:1000 in PBS for 2 or 4 h caused cell viability to decrease by almost 50% (**Figure 5A**). Therefore, we exposed primary NK cells to ASGM-1 with dilution of 1:1000 in PBS for 4 h to establish a NK-cell depletion model *in vitro*, and NK cells were treated with SM simultaneously. As shown in **Figure 5B**, the addition of SM partially but significantly reversed the depletion of NK cell with the increasing expression of NKG2D and ratio of NKG2D/Ly49A (**Figure 5C**).

### SM Enhanced NK-Cell Activation on Inhibiting HSCs *in Vitro*

In order to check if SM could enhance the activation of NK cells inhibiting HSCs *in vitro*, NK cells were pre-incubated with or without SM for 8 or 16 h. Then the cells were collected and the culture supernatants were remained. JS-1 cells were firstly co-cultured with or without the pre-incubated NK cells at ratio of 50:1 (NK cell: HSC = 50:1) for 5 h. Since NK cells were activated when expression of ligand for NK cell receptors were altered on the target cells, RAE-1ε, the NK cell stimulatory ligand on the surface of HSCs, was observed. As illustrated in **Figures 5D,E**, expressions of RAE-1ε on JS-1 cells were up-regulated while levels of α-SMA were down-modulated after co-cultured with the NK cells. Surface expression of RAE-1ε on HSCs were significantly elevated by the pre-incubated NK cells which were pre-treated with SM for 16 h. To further investigate the effects of SM on NK-cell activation in inhibiting activities of HSCs, the previous collected culture supernatants were added into the adherent JS-1 cell with another period of 5 h. Later, expressions of α-SMA and RAE-1ε on HSCs were also assayed. As shown in **Figures 5F,G**, the culture supernatants in which the NK cells had been pre-incubated with SM could also decrease the levels of α-SMA and increase the expression of RAE-1ε on HSCs. Taken together, these data suggested that the functions of NK cells on inhibiting activation of HSCs could be enhanced after SM treatment *in vitro*.

## Discussion

Chronic liver diseases, such as liver cirrhosis, virus hepatitis, liver cancer and autoimmune hepatitis, affect billions of people worldwide, which are initially associated with liver fibrosis. During the process of liver fibrosis, HSCs play a key role in fibrogenesis, and differentiate into myofibroblast-like cells that are proliferative and fibrogenic upon activation following liver injury (Tsuchida and Friedman, 2017). Huge amounts of studies were mainly focused on HSCs, however, more and more researches have shown that the formation of liver fibrosis involves complex immunopathological mechanisms. The interactions between HSCs and hepatic immune cell subsets have emerged as important determinants of liver fibrosis progression and regression (Hernandez-Gea and Friedman, 2011). It is accepted commonly that T cells, B cells and NK T cells play dual roles in controlling HSC activation and liver fibrosis by produce both anti-fibrotic and pro-fibrotic cytokines (Wynn, 2004; Holt et al., 2006; Park et al., 2009). Particularly, NK cell could kill activated HSCs, which was confirmed by many subsequent researches both in animal models (Jeong et al., 2006; Radaeva et al., 2007; Gur et al., 2012) and patients (Muhanna et al., 2011; Gur et al., 2012). The underlying mechanisms of the interactions between NK cells and HSCs have been investigated widely in recent years, but the mechanism still remains to be unclearly. There is still an urgent need to uncover effective medicine reversing fibrosis to prevent disease progression.

Due to an increasing interest in alternative medicine, many researchers have attempted to elucidate the mechanism underlying the activities of traditional Chinese medicine. SM, as a standard herb for invigorating blood circulation and eliminating stasis in TCM, plays a crucial role in regulating the disorder of blood stasis and used effectively in clinical practices for 100s of years. Accumulating evidence from both animal and human studies indicates that SM is capable of modulating the liver injury, especially reversing liver fibrosis. But the anti-fibrotic mechanism of SM in regulating the immune disorder is not well-defined. Therefore, the aim of the present study was to investigate the effects of SM on regulating the immunologic functions of hepatic NK cells during liver fibrosis.

In this study, we found that SM treatment significantly alleviated serum ALT and AST, and reduced liver inflammation and fibrosis in the CCl_4_-induced model. Remarkably, the anti-fibrogenic effects of NK cell were reportedly suppressed during advanced liver injury (Jeong et al., 2011). We found the effects of SM treatment in CCl_4_-induced liver fibrosis was correlated with the increased hepatic NK activity, which was manifested through the elevated frequency of hepatic NK cells, since NKG2D and NKp46, the activation receptors of NK cells, were apparently up-regulated by SM treatment.

Since hepatic NK cells were sharply reduced in the liver fibrosis model and could be significantly excited by SM treatment, we wondered whether SM could affect the activities of NK cells *in vitro*. If so, we even wondered whether SM could strengthen the activities of NK cells on inhibiting the activation of HSCs. To decipher the contribution of SM to the activation of liver NK cells, primary hepatic NK cells were isolated and were then incubated with SM *in vitro*. Synchronously, JS-1 cells were also incubated with different concentration of SM. Collectively, we opted to find the compatible drug concentration for SM to driving the activities of NK cells as well as to suppressing the activation of HSCs. After preliminary screening the toxicity of SM on these two types of cells, we found 50 μg/ml of SM incubation led to no obvious toxic to HSCs, but significantly promoted the survival ratio of NK cells. Subsequently, JS-1 cells were activated with TGF-β1 and the anti-fibrotic effects of SM treatment were testified with immunofluorescence staining of F-actin, α-SMA and Collagen I. We then tested the expressions of NKG2D, IFN-γ, trail and perforin of NK cells after SM-incubation. Although expressions of trail and perforin were not augmented, the levels of NKG2D and IFN-γ were significantly increased after SM treatment *in vitro*. These results indicated that SM could promote activities of primary NK cells and inhibited HSC activation *in vitro*.

Moreover, to further confirm the enhancement effect of SM on NK cells, we opted to deplete NK cells by the ASGM-1 antibody. ASGM-1 is a rabbit polyclonal antibody that reacts with a neutral glycosphingolipid expressed on the surface of numerous hematopoietic cells including NK cells, NK T cells, CD8^+^T cells, γδ T cells, some CD4^+^T cells, macrophages, eosinophils, and basophils (Trambley et al., 1999; Slifka et al., 2000; Nishikado et al., 2011). Since ASGM-1 effectively eliminates only NK cells and basophils *in vivo*, which indicated that ASGM-1 was one of the most precise tools available to specifically eliminate NK cells, we used anti-ASGM-1 antibody to deplete the activities of NK cells *in vitro*. We wondered whether the depletion of NK cells would lead to a diminished anti-fibrotic therapeutic effect of SM. Therefore, we investigated the effect of SM on ameliorating the suppressive vitality of primary hepatic NK cells. After 4 h incubation, ASGM-1 significantly inhibited the viability of NK by almost 50% when compared with that of the control. And SM enhanced the viability of NK by nearly 30%. NKG2D is the stimulatory receptors interacting with the stimulatory ligand RAE-1 which is expressed on the surface of the HSCs and promote NK cell activities. And Ly49A is the inhibitory receptor for NK cells. Here, we assayed the expression of NKG2D and Ly49A on NK cells by RT-qPCR to evaluate the improvement of SM on NK cells. After incubation with SM for 2 or 4 h, the expressions of NKG2D were up-regulated in a time-dependent manner.

SM inhibiting HSCs activation has been confirmed in animal studies (Wasser et al., 1998; Sferra et al., 2012; Zhang et al., 2013). During the preparation and identification of the drug, we also find that SM contains many ingredients, such as tanshinol, salvianolic acid B, dihydrotanshino, cryptotanshinon, and tanshinone IIA, etc. This also suggests that the mechanism of SM exerting antifibrotic effect is not unique. In addition to directly inhibiting HSCs activation, promoting NK cell activity might also be one of the anti-fibrotic mechanisms for SM. Thus, hepatic NK cells were pre-incubated with SM for 8 or 16 h before they were co-cultured with JS-1 cells in order to explore the enhancement of SM on NK-cell activation in inhibiting HSCs *in vitro*. After co-culture with or without the pre-incubated NK cells or the culture supernatants, expression of the NK cell stimulatory ligands on HSCs for NK cell receptors were assayed. As expected, levels of α-SMA and RAE-1ε on HSCs were diminished after the HSCs were co-cultured with the pre-incubated NK cells and their culture supernatants. After 16 h co-culture with pre-incubated NK supernatant or NK cells, the expression of α-SMA in HSCs decreased sharply by nearly 2.5 times with the NK only group. In summary, these data indicated that the activities of NK cells on inhibiting HSCs were significantly boosted after the pre-incubation with SM, which confirmed that the anti-hepatic effect of SM is partly achieved by enhancing the activity of NK cells.

## Conclusion

SM was demonstrated to be effective for alleviating liver fibrosis induced by CCl_4_ via promoting activities of hepatic NK cells *in vivo* and *in vitro*.

## Author Contributions

YP performed the experiments. YP and YT analyzed the data and wrote the manuscript. TY identified the drug. KH assisted with the animal experiment. LS assisted with the cell culture. CL made critical revision for the manuscript. YT and CL co-correspond for the whole project.

## Conflict of Interest Statement

The authors declare that the research was conducted in the absence of any commercial or financial relationships that could be construed as a potential conflict of interest. The reviewer XT and handling Editor declared their shared affiliation.
